# Involvement of Seladin-1 in goniothalamin-induced apoptosis in urinary bladder cancer cells

**DOI:** 10.1186/1472-6882-14-295

**Published:** 2014-08-09

**Authors:** Heng Kai Yen, Afifah-Radiah Fauzi, Laily Bin Din, Valerie J McKelvey-Martin, Chan Kok Meng, Salmaan Hussain Inayat-Hussain, Nor Fadilah Rajab

**Affiliations:** Biomedical Science Programme, Faculty of Health Sciences, Universiti Kebangsaan Malaysia, Jalan Raja Muda Abdul Aziz, 50300 Kuala Lumpur, Malaysia; Faculty of Science and Technology, Universiti Kebangsaan Malaysia, Bangi, 43600 Selangor Malaysia; School of Pharmacy and Pharmaceutical Sciences, University of Ulster, Coleraine, BT52 1SA United Kingdom; Environmental Health and Industrial Safety Science Programme, Faculty of Health Sciences, Universiti Kebangsaan Malaysia, Jalan Raja Muda Abdul Aziz, 50300 Kuala Lumpur, Malaysia; Toxicology Laboratory, Faculty of Health Sciences, Universiti Kebangsaan Malaysia, Jalan Raja Muda Abdul Aziz, 50300 Kuala Lumpur, Malaysia

**Keywords:** Seladin-1, Bladder cancer, Goniothalamin, Apoptosis

## Abstract

**Background:**

Selective Alzheimer Disease Indicator-1 (or Seladin-1) is a multifunctional protein first discovered by downregulation of its expression in Alzheimer’s disease. Interestingly, the expression of this protein is upregulated in several cancers, including primary bladder cancer. However, its role in cancer formation has yet to be discovered. Goniothalamin is a natural product that has been demonstrated to induce apoptosis in various cancer cell lines. In this study, we have elucidated the role of Seladin-1 in goniothalamin-induced cytotoxicity towards human urinary bladder cancer cell line RT4.

**Methods:**

The cytotoxicity of goniothalamin in human urinary bladder cancer cell line RT4 was assessed using MTT assay and the mode of cell death was determined by Annexin V-FITC/PI labeling assay. Finally, the expression of Seladin-1 protein in goniothalamin-treated RT4 cells was determined by Western blot.

**Results:**

MTT assay showed that the cytotoxicity of goniothalamin on RT4 cells was concentration and time dependent with IC_50_ values of 61 μM (24 hr), 38 μM (48 hr) and 31 μM for 72 hr, respectively. Cell death induced was confirmed through apoptosis; as assessed using the Annexin V-FITC/PI labeling assay. Furthermore, the involvement of Seladin-1 in goniothalamin-induced apoptosis was evidenced through the cleavage of 60 kDa protein to 40 kDa and 20 kDa. This was followed by a gradual increase of 20 kDa fragment suggesting the involvement of Seladin-1 in goniothalamin-induced apoptosis on RT4 cells.

**Conclusion:**

This study demonstrates that goniothalamin induce cytotoxicity and apoptosis on RT4 cells. The involvement of Seladin-1 in goniothalamin-induced apoptosis further suggested that Seladin-1 may play a role in the formation of primary bladder cancer.

## Background

The urinary bladder functions as a temporary storage for urine before being excreted out from the body. It is composed of layers of transitional epithelium; including smooth muscle, which allows the organ to expand and contract [1]. It is an organ highly prone to cancer of genitourinary system, with 90% of the cancer being transitional cell carcinoma, whilst the rest are squamous and adenocarcinoma [2]. The etiology of urinary bladder cancer include tobacco smoking, occupational exposure to chemical carcinogens, such as aromatic amine and Schistosomal infections [3, 4]. The progression of cancer is a multistep process that includes genetic alteration; as the deregulation (or mutation) of certain genes may predispose to certain cancers. According to Doherty and coworkers, different gene expressions that were found at two anatomical areas (ureteric orifice and dome) of normal bladders are commonly prone to have primary bladder cancer. The expressions of these genes may give some insight into bladder carcinogenesis; especially Seladin-1, which was found upregulated at the ureteric orifice. However, little is known about this gene on its involvement especially in cancers [5–7].

Selective Alzheimer’s disease Indicator 1 (or Seladin-1) was first discovered in Alzheimer’s disease. This gene has an anti-apoptotic function in brain tissue, whereby its downregulation has been associated with neurodegeneration [8]. Seladin-1 was found to be identical to the gene DHCR24, which functions as a key enzyme in cholesterol biosynthesis and lipid raft formation [9, 10]. It was also described as a multifunctional protein. In cancer, Seladin-1 was found highly expressed in adrenal cancer, pituitary tumors, urinary bladder cancer, melanoma, and prostate cancer [7, 11–14]. The high expression of Seladin-1 found in those cancers may have been due to high cholesterol content; however, the significance of Seladin-1 in cancer was not fully elucidated. From previously reported literature, we strongly believe in the involvement of Seladin-1 in carcinogenesis. It has also been shown that Seladin-1 was cleaved by caspase-6 and caspase-3, which proved Seladin-1 act as a death substrate for caspases and may function as pro-apoptotic element upon cleavage [8]. According to previous studies, upon oncogenic induction and oxidative stress, Seladin-1 was found to bind p53 and displace mdm-2 resulted in the accumulation of active p53. This interaction suggests a role of Seladin-1 in the regulation of cell fate [15]. In addition, Seladin-1 was demonstrated as lipopolysaccharide (LPS) responsive gene product that regulates anti-inflammatory response [16].

Goniothalamin is a natural product isolated from Goniothalamus species, which is abundantly found in Malaysia. This styryl lactone derivative possesses potent cytotoxic activity and induces apoptosis in various cancer cell lines; such as liver, breast, kidney, ovarian and leukemic cell lines [17–23]. Recent studies have demonstrated the anti-inflammatory and antiproliferative effect of goniothamin *in vivo* to further confirm the potential of goniothalamin and its related molecules as a therapeutic agent [24]. The mechanism of goniothalamin-induced apoptosis was found involves oxidative stress and mitochondria mediated pathway [17, 25]. Previous studies have shown that goniothalamin induces DNA damage, generation of reactive oxygen species (ROS), loss of mitochondria membrane potential, release of cytochrome c and activation of caspases [17, 18, 21, 22, 25]. Goniothalamin-induced apoptosis mainly via intrinsic pathway; however, the full mechanism is yet to be elucidated. Thus, in this study, the cytotoxicity of goniothalamin was assessed using MTT assay in urinary bladder cancer cell line RT4 and etoposide, a known cytotoxic agent was used as a positive control. The determination of apoptosis event and the involvement of Seladin-1 in goniothalamin-induced apoptosis were also elucidated, to further understand the underlying mechanism.

## Methods

### Goniothalamin

The compound goniothalamin was provided by Professor Dato Dr. Laily Bin Din from Universiti Kebangsaan Malaysia (Bangi). It was isolated from *G. andersonii* as described previously [17, 26], a stock solution of 50 mM goniothalamin was prepared by dissolving in DMSO (Ajax Finechem) with the final concentration less than 1%.

### Cell line & reagents

Urinary bladder cancer cell lines RT4 was obtained from ATCC and cultured in McCoy’s 5A medium (Gibco) supplemented with 10% fetal bovine serum and 1% penicillin/streptomycin. Cells were subcultured every two days with 0.25% trypsin/ 1.5 mM EDTA and maintained at 37°C under 5% CO_2_ environment. The RT4 cells used in this study were within 10 passages.

### MTT assay

The cytotoxicity of goniothalamin on RT4 cells was assessed by MTT assay as described previously [25, 27]. RT4 cells were seeded in a 96 well microplate at a concentration of 5 × 10^4^ cells per well and incubated 24 hrs. Next, a fresh medium containing tested compound was added. The concentrations of goniothalamin used were 6.25, 12.5, 25, 50 and 100 μM and etoposide were 18.75, 37.5, 75, 150 and 300 μM. Cells were treated for 24, 48 and 72 hrs. After treatment, 20 ul MTT (5 mg/ml) was added and further incubated for 4hrs. Supernatant was then discarded and replaced with 200ul DMSO. The microplate was incubated for 15m and shaken to dissolve the crystal formazan. The viability of the cells was determined by measuring the absorbance of each well on an ELISA plate reader (Biorad) at 570 nm. The percentage viability of the cells was determined by comparing the mean absorbance of treated cells to untreated cells. A graph was plotted with percentage viability versus concentration of the tested compound and IC_50_ value was obtained.

### Annexin V-FITC/PI labeling assay

Determinations of the mode of cell death or the apoptosis event induced by the goniothalamin on RT4 cells were assessed by Annexin V-FITC/PI labeling assay, as described previously [25]. RT4 cells were seeded at 1 × 10^6^ cells per well in a six well plate and incubated for 24 hrs, followed by treatment of goniothalamin at concentrations of 50, 61 (IC_50_) and 100 μM, and treatment of etoposide at 46 μM (IC_50_) act as positive control, and incubated for 24hrs. After treatment, cells were harvested. Medium and floaters from each well were collected and cells were rinse with PBS and incubated with trypsin/EDTA until 80% of the cells were detached. All cell suspensions were collected and centrifuged at 220 × *g* for 5 mins. Next, the supernatant was discarded and the pellets were washed with chilled PBS and centrifuged at 220 × *g* for 5 mins at 4°C. Pellets were washed twice and the supernatant was discarded. Next, 150 μl of chill Annexin V binding buffer was added, followed by 5 μl of Annexin V-FITC (BD Pharmingen) and incubated for 15 mins. During the final 2 mins, 5 μl of propidium iodide (50 μg/ml) was added and further incubated. After staining, 350 μl of Annexin V binding buffer was added and all of the samples were transferred to Falcon flow tubes for analysis using FACSCanto Flow Cytometer (BD Bioscience, USA).

### Western blotting

RT4 cells were seeded at a concentration of 2.5 × 10^5^ cells/ml on a tissue culture dish and incubated for 24 hrs. Next, the cells were treated with goniothalamin at the concentration of 61 μM (IC_50_) for 2, 4, 8, 16, and 24 hrs. Following treatment, cell lysates were collected and protein samples were prepared and denatured at 95°C for 5 mins. Next, 20 μg/ml lysate was resolved on 12% SDS-polyacrylamide gel electrophoresis and blotted onto a polyvinylidene fluoride membrane. Western blotting was carried out as described previously [18]. Rabbit monoclonal anti-Seladin-1 antibody (Sigma, USA) was used at a dilution of 1:5000 in 5% skimmed milk and incubated at 4°C overnight. Meanwhile, rabbit monoclonal beta actin antibody (Cell Signaling, USA) was used at a dilution of 1:10000. After incubation of the primary antibody, secondary antibody (goat anti-rabbit HRP conjugated antibody, Cell Signaling, USA) was added and incubated for 1 hr. prior to detection by chemiluminenscene (Amersham, UK).

### Statistical analysis

All experiments were repeated three times and data were presented as mean ± SEM. Statistical analysis was performed using Statistical Package for Social Science (SPSS) version 21 and One Way ANOVA post-hoc technique was used. A *p* value < 0.05 was considered statistically significant.

## Results

### MTT assay

**Figure 1:**
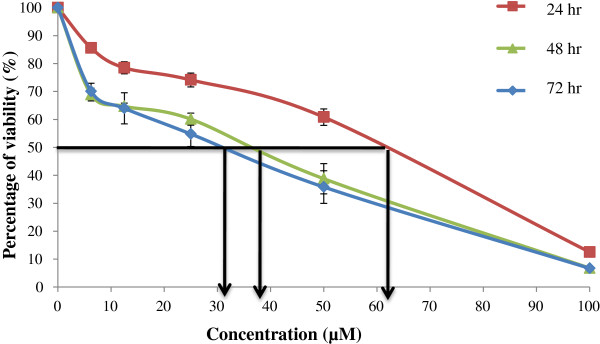
**Percentage of viability of RT4 cells treated by goniothalamin (6.25, 12.5, 25, 50 & 100 μM) for 24, 48 & 72 hr.** Each point represents mean ± SEM of 3 different experiments (*p < 0.05 compare to control).

**Figure 2:**
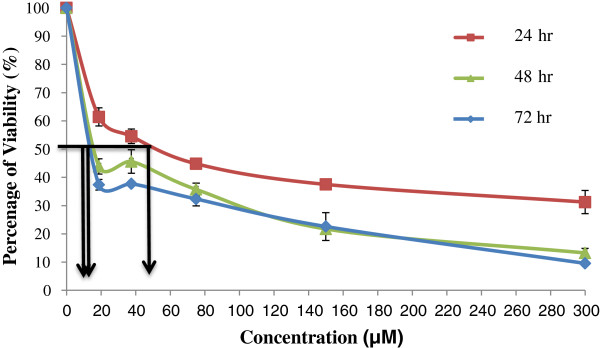
**Percentage of viability of RT4 cells treated by etoposide (18.75, 37.5, 75, 150, and 300 μM) for 24, 48, and 72 hrs.** Each point represents mean ± SEM of 3 different experiments (*p < 0.05 compared to the control).

The cytotoxic effect of goniothalamin on RT4 cells was assessed by MTT assay. Following treatment of 24, 48 and 72 hr, the percentage viability of the cells was obtained and graph was plotted versus concentration of goniothalamin (see Figure 1). The graph shows that the IC_50_ value was 61 μM, 38 μM and 31 μM following 24 hrs, 48 hrs and 72 hrs treatment, respectively. The viability drop from 100% from control and further reduced across concentrations of goniothalamin and time points; with the highest concentration (100 μM) of goniothalamin showed less than 20% of cells were viable following 24 hrs, 48 hrs, and 72 hrs of treatment. Statistical analysis showed that there was a significant reduction in the percentage of cell viability following treatment with goniothalamin at each concentration (i.e., 6.25, 12.5, 25, 50, and 100 μM) compared to control or untreated cells (*p* < 0.05). Etoposide was used as a positive control for this experiment and the results of the cytotoxic effect are shown in Figure 2. The IC_50_ value of etoposide on RT4 cells was 46 μM, 15 μM and 14 μM following 24 hrs, 48 hrs and 72 hrs treatment, respectively. There was a significant reduction in percent viability compared to the control at each concentration of etoposide (*p* < 0.05).

### Annexin V-FITC/PI labeling

**Figure 3:**
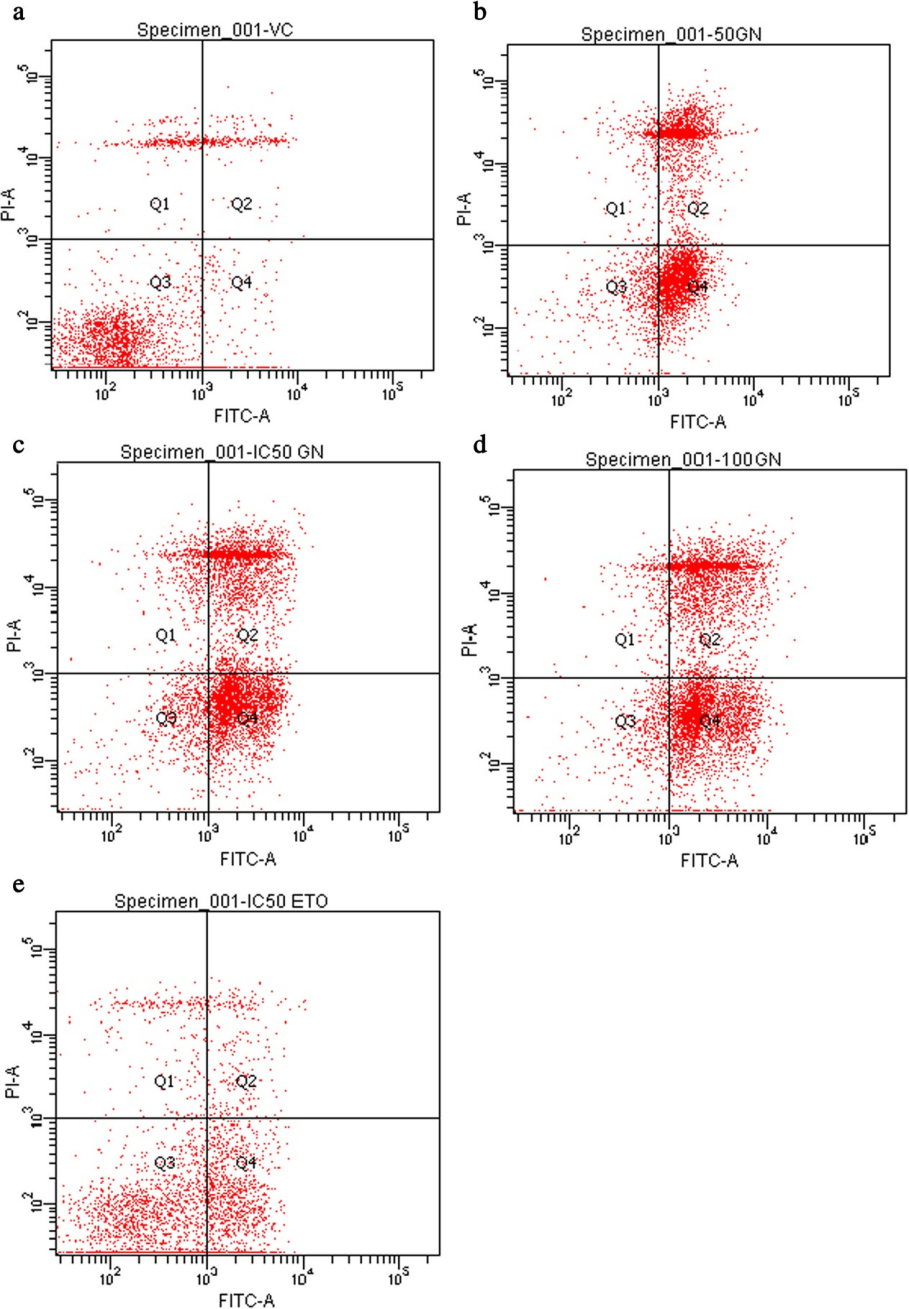
**Flow cytometry profile of RT4 cells treated by goniohtalamin (GTN) at 50, 61.3 (IC**_**50**_ **), and 100 μM, etoposide IC**_**50**_ **for 24 hrs., a) Untreated cells (VC) b) GTN 50 μM; c) IC**_**50**_ **GTN d) 100 μM GTN and e) etoposide IC**_**50**_ **.**

Goniothalamin was known to induce cell death with mainly through apoptosis at various cell lines [17–23]. In this study mode of cell death or determination of apoptosis event induced by goniothalamin on bladder cancer cell line RT4 was assessed by using Annexin V-FITC/PI Labeling assay as described previously [25], at the concentration of 50, 61 (IC_50_) &100 μM of goniothalamin following 24 hr treatment. Three concentration of goniothalamin was chosen in order to understand the concentration dependent cell death event induced by goniothalamin. By using Annexin V-FITC/PI staining, apoptosis and necrosis event was quantified by flow cytometry as shown in Figure 3. The cytogram quadrant of annexin V staining positive represent apoptotic event and PI staining positive represent necrotic event. Quadrant 1 (Q1) is the region of necrotic event where annexin V staining negative and PI staining positive. Q2 is late apoptosis event where both annexin V and PI staining positive, Q4 is early apoptosis event with cells stained with annexin V alone and Q3 represent event of negative staining for both annexin V and PI. Based on the result, there was an increased in the apoptosis event with the increase of goniothalamin concentration. At 50 μM, 53.6% of the cells are apoptotic, whereas 63.8% are apoptotic at 61μM treatment and 70.5% at 100 μM, respectively. However, etoposide showed only 25.1% of apoptosis event. Only small percentages of cells were necrotic. Vehicle control or untreated cell was also assessed to ensure the viability of cell for each experiment, majority all the event fall into Q 3 which was 81.9% of cell stained negative for both stain. Statistical analysis showed significant apoptosis event compare to untreated cells with p < 0.05, with no significant difference for necrotic events.

### Western blotting

**Figure 4:**
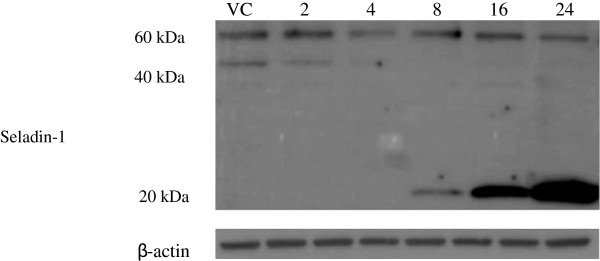
**Expression of Seladin-1 on RT4 cells after treatment by IC**_**50**_ **(61 μM) of goniothalamin at 2, 4, 8, 16, and 24 hrs.** Vehicle control (or untreated cells) acted as a negative control and β-actin as a loading control during the experiment.

The involvement of Seladin-1 protein on induction by goniothalamin was assessed using Western blotting (as shown in Figure 4). RT4 cells were treated by IC_50_ of goniothalamin at 2, 4, 8, 16, and 24 hrs to assess the expression of Seladin-1. The results showed that there was a cleavage of Seladin-1 from 60 kDa to 40 kDa, and 20 kDa as increase in treatment time, and the 40 kDa fragment was gradually decreased but not visualized at a later time point. On the contrary, the 20 kDa fragment was gradually increased from 8, 16, and 24 hours.

## Discussion

### Goniothalamin induced cytotoxicity and antiproliferative on RT4 cells

Goniothalamin is a styryl lactone derivative that has been proved to have cytotoxic activity and induce apoptosis in a variety of cancer cell lines [28]. However, the cytotoxicity of goniothalamin on urinary bladder cancer has not been assessed before. Therefore, in this study, urinary bladder cancer cell line RT4 was employed to determine the cytotoxicity of goniothalamin on urinary bladder cancer. RT4 cells were treated by goniothalamin 6.25, 25, 50, and 100 μM for 24 hrs, 48 hrs, and 72 hrs. Results showed that IC_50_ for 24 hrs was 61 μM; which was higher than previous studies [23, 28]. Therefore, the treatment time was extended to 48 hrs and 72 hrs; where IC_50_ for 48 hrs treatment was 38 μM and 31 μM for 72 hrs respectively. According to previous studies, goniothalamin was able to induce cytotoxic and apoptosis at micro molar concentrations; Chan and coworkers have demonstrated that the IC_50_ value for 72 hr treatment was 22 μM on vascular smooth muscle cells [28]. This result and our findings indicate that goniothalamin is able to induce concentration and time dependent cytotoxic and antiproliferative effects; the effect of which may depend on sensitivity, stage, and type of cancer. An RT4 cell has the characteristic similar to superficial bladder tumor with bearing wild type p53 [29]. According to Al-Sukhun and coworkers, aberration of tumor suppressor gene such as p53 was associated with invasive cancer phenotype and influenced the responsiveness of the tumor to chemotherapeutic agents [30]. This may explained the higher IC_50_ value in this study.

### Goniothalamin induced apoptosis on RT4 cells

Apoptosis was assessed using Annexin V-FITC/PI staining. The assay utilized Annexin V, which is a protein that binds to phosphatidylserine (PS) when cells undergo apoptosis. The externalization of PS was an early event of apoptosis; thus, by using annexin V, conjugated with a fluorochrome (FITC), the event of apoptosis could be quantified by flow cytometer. Based on the results obtained, goniothalamin was able to induce apoptosis on RT4 cells after 24 hrs treatment. Event of apoptosis were concentration dependent, where treatment with 100 μM of goniothalamin gave the highest apoptosis event (70.5%). When comparing IC_50_ of etoposide, 46 μM to IC_50_ of goniothalamin, events of apoptosis were higher in goniothalamin. This may have been due to different end points of each assay used to detect cell viability and apoptosis, as MTT assay only measured the activation of the mitochondria enzyme [27], while annexin v-FITC labeling was able to detect the flipping of PS on the apoptosis cells [31]. However, in some incidents, the flip-flop of PS may also have occurred as a late event; and in this research, etoposide only acted as a positive control for experiment validation [32]. In addition, necrotic events induced by goniothalamin were less than 10%, which similar to previous studies on various cancer cell lines including cervical cancer, leukemia, vascular smooth muscle cells, and breast cancer cells [23, 25].

### Involvement of Seladin-1 in goniothalamin induced apoptosis

Previous studies have shown that styryl lactone compound from *Goniothalamus sp* was able to induce apoptosis via activation of caspases enzyme cascade, release of cytochrome c, loss of mitochondrial membrane potential, and increase expression of pro-apoptotic BH3 protein [33]. Therefore, goniothalamin was used in this study as apoptotic inducer to elucidate the role of Seladin-1 in goniothalamin induced apoptosis on human bladder cancer cell line RT4. Upon treatment with goniothalamin, we observed that Seladin-1 was cleaved from 60 kDa to 40 kDa, and 20 kDa. As increase in treatment time, the 40 kDa fragment was undetectable. However, the 20 kDa showed a gradual increased. This result was comparable to previous studies by Greeve and coworker, which showed that upon induction of apoptosis by growth factor deprivation, Seladin-1 was cleaved by caspase-3 and caspase 6 to 4 multiple fragments of 20, 30, 40, and 50 kDa on human umbilical vein endothelial cells [8]. However, in this study, instead of a 40 kDa fragment, goniothalamin treatment was able to induce a cleavage of Seladin-1 to 20 kDa on RT4 cells. This cleavage was believed to be mainly due to the activation of caspase-3. In accordance with previous studies, caspase-3 was the primary executioner of caspases activated during goniothalamin-induced apoptosis [17, 18, 25]. Therefore, in this study, we are in agreement with Greeve and coworker that Seladin-1 was a caspase-3 substrate and following the induction of apoptosis by goniothalamin, Seladin-1 was believed to be cleaved and the expression increased; which may resulted in changes of function from anti-apoptotic to pro-apoptotic (as shown in this study). In addition, this evidence also suggested that Seladin-1, which function as anti-apoptotic protein, may play a role in the formation of primary bladder cancer. Hence, Seladin-1 can act as a therapeutic target for urinary bladder cancer, or as a screening marker for primary tumors.

## Conclusion

Goniothalamin induced cytotoxicity through apoptosis in human urinary bladder cancer cell line RT4, in concentration and time dependent manner. The involvement of Seladin-1 was observed with increased expression of cleaved fragment (20 kDa) further suggested its role in goniothalamin-induced apoptosis and formation of primary cancer. However, further investigation is needed to elucidate the role of Seladin-1 in urinary bladder cancer.
